# Rosmarinic Acid, Active Component of Dansam-Eum Attenuates Ototoxicity of Cochlear Hair Cells through Blockage of Caspase-1 Activity

**DOI:** 10.1371/journal.pone.0018815

**Published:** 2011-04-15

**Authors:** Hyun-Ja Jeong, Youngjin Choi, Min-Ho Kim, In-Cheol Kang, Jeong-Han Lee, Channy Park, Raekil Park, Hyung-Min Kim

**Affiliations:** 1 Biochip Research Center, Hoseo University, Asan, Chungnam, Republic of Korea; 2 High-Enthalpy Plasma Research Center, Chonbuk National University, Jeonju, Republic of Korea; 3 Vestibulocochlear Research Center and Department of Microbiology, Wonkwang University School of Medicine, Jeonbuk, Republic of Korea; 4 Department of Audiology, Nambu University, Gwangju, Jeonnam, Republic of Korea; 5 Department of Pharmacology, College of Oriental Medicine, Kyung Hee University, Seoul, Republic of Korea; University of Muenster, Germany

## Abstract

Cisplatin causes auditory impairment due to the apoptosis of auditory hair cells. There is no strategy to regulate ototoxicity by cisplatin thus far. Dansam-Eum (DSE) has been used for treating the central nerve system injury including hearing loss in Korea. However, disease-related scientific investigation by DSE has not been elucidated. Here, we demonstrated that DSE and its component rosmarinic acid (RA) were shown to inhibit apoptosis of the primary organ of Corti explants as well as the auditory cells. Administration of DSE and RA reduced the thresholds of the auditory brainstem response in cisplatin-injected mice. A molecular docking simulation and a kinetic assay show that RA controls the activity of caspase-1 by interaction with the active site of caspase-1. Pretreatment of RA inhibited caspase-1 downstream signal pathway, such as the activation of caspase-3 and 9, release of cytochrome *c*, translocation of apoptosis-inducing factor, up-regulation of Bax, down-regulation of Bcl-2, generation of reactive oxygen species, and activation of nuclear factor-κB. Anticancer activity by cisplatin was not affected by treatment with RA in SNU668, A549, HCT116, and HeLa cells but not B16F10 cells. These findings show that blocking a critical step by RA in apoptosis may be useful strategy to prevent harmful side effects of ototoxicity in patients with having to undergo chemotherapy.

## Introduction

Cisplatin is a highly effective and widely used anticancer agent [1]. The risk of ototoxic and nephrotoxic side effects commonly hinders the use of higher doses that could maximize its antineoplastic effects [2]. Cisplatin has been shown to induce auditory sensory cell apoptosis [3]–[5]. Devarajan et al. recently reported cisplatin-induced apoptosis in an immortalized cochlear cell line [6]. Cisplatin toxicity also was associated with an increase in caspase-3, caspase-8, and caspase-9 activity, cytochrome *c* release, apoptosis-inducing factor (AIF) translocation, reactive oxygen species (ROS) generation, and nuclear factor-κB (NF-κB) activation [7], [8].

Caspase-1 is a member of the cystein-aspartic acid protease (caspase) family [9]. Caspase-1 is characterized by its ability to activate the inactive precursors of interleukin (IL)-1β and IL-18, cytokines that are involved in inflammation. It contains an N-terminal caspase recruitment domain (CARD). CARD promotes proteolytic activation of recruited caspases in apoptosis and inflammation [10].

Given that hair cells do not regenerate in the mammalian cochlea, cell loss, e.g., due to noise, hypoxia, or cisplatin, is irreversible and cumulative [4], [11]. Cisplatin primarily damages the outer hair cells (OHCs) of the organ of Corti, which are the specific effectors of mammalian cochlear amplification and frequency determination. Larger doses of cisplatin are associated with additional damage to auditory neurons, stria vascularis, and supporting cells near the OHCs [12]. Therefore, selective inhibition of these pathways may provide a strategy to minimize cisplatin-induced ototoxicity.

Dansam-Eum (DSE) has been used to treat stagnation in the upper area of the body in Korean Medicine. In particular, DSE has been used to cure hearing problems that are collectively expressed as the ‘deficiency syndrome’. Vessel occlusion and cochlear blood hypo flow are the major cause of noise-induced hearing loss and idiopathic sudden sensorineural hearing loss. DSE activates blood and dissipates blood stasis; it promotes blood flow and removes static blood in the treatment of blood stasis [13]. Rosmarinic acid (RA), a water-soluble polyphenolic component isolated from DSE, has been reported to have anti-oxidative [14], anti-inflammatory [15], and anti-depressive activities [16]. Li et al. reported that RA was found in the brain following intravenous administration of *Salvia miltiorrhiza* [17]. Hence, RA can be considered as an active compound that improves the health of auditory regulation organs. The aim of this study is to explore the effect and mechanism of RA on hearing loss.

## Results

### RA inhibits apoptosis

**Figure 1:**
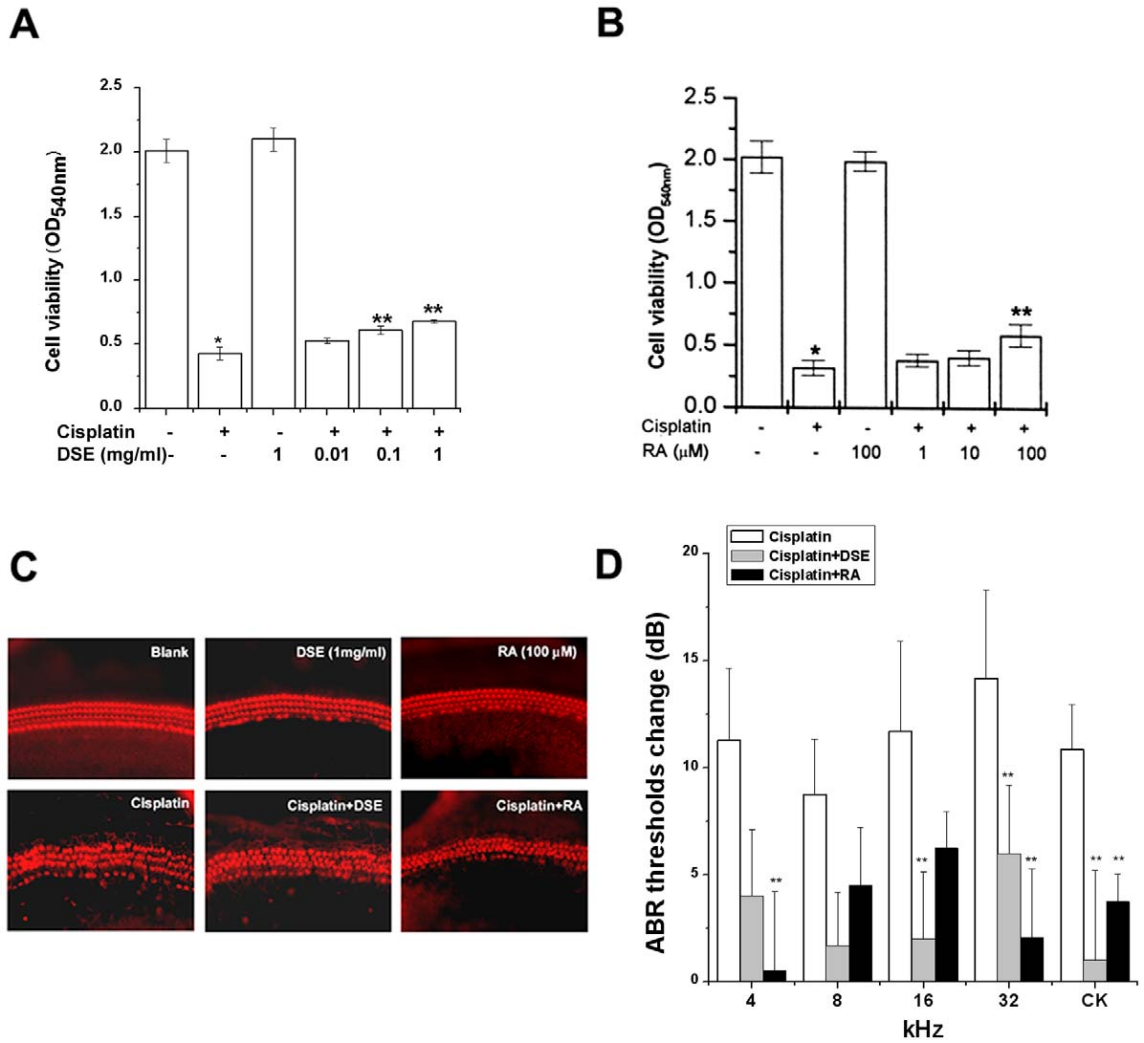
DSE or RA inhibits apoptosis. HEI-OC1 cells (1×10^5^/well) were treated with various concentrations of DSE (0.01, 0.1, and 1 mg/ml) or RA (1, 10, and 100 µM) for 4 h and then stimulated with cisplatin (20 µM) for 48 h. Cell viability was estimated by MTT assay (A and B). The organ of Corti explants were treated with media, 20 µM cisplatin, 1 mg/ml DSE, 100 µM RA, cisplatin plus 1 mg/ml DSE, and cisplatin plus 100 µM RA for 30 h. The organ of Corti explants were stained with TRITC conjugated phalloidin, and then observed under fluorescent microscope (C). C57BL/6 mice (each group, *n* = 9) were given orally administration of DSE (1 g/kg) or intraperitoneal injections of RA (4 mg/kg) before cisplatin for four consecutive days (4 mg/kg body weight per injection). ABR was examined as described in the methodology (D). Results are representative of three independent experiments. Data are mean ± S.D. of three independent experiments performed in duplicate. *P<0.01, compared with unstimulated cells (determined by independent *t*-test). **P<0.05 compared with cisplatin alone (determined by ANOVA analysis).

To determine the effects of DSE, RA (a component of *Salvia miltiorrhiza* Bunge) or tanshinnone IIA (a component of *Salvia miltiorrhiza* Bunge) on cell viability, HEI-OC1 cells were exposed to 20 µM cisplatin in combination with an increased concentration of DSE, RA, or tanshinnone IIA. The effect of DSE, RA, or tanshinnone IIA was initially assessed on viability of HEI-OC1 cells using 3-[4, 5-dimethylthiazol-2-yl]-2, 5-diphenyl- tetrazolium bromide (MTT) assay. As shown Fig. 1A and B, when the cells were treated for 48 h with cisplatin, the cell viability decreased significantly compared with media control. DSE or RA inhibited cisplatin-induced cell death of HEI-OC1 cells. But tanshinnone IIA did not inhibit cisplatin-induced cell death (data not shown). This indicates that RA is an active compound of DSE.

To examine whether DSE or RA protects the primary organ of Corti explants from cisplatin, the half middle turn of the organ of Corti from neonatal (P2) Sprague Dawley rats was isolated and treated with DSE or RA and cisplatin for 30 h. Tetramethylrhodamine isothiocyanate (TRITC)-conjugated phalloidin, which binds to F-actin (polymeric fibrous actin), was used to stain hair cells, Hensen's cells, and Claudius cells in the cochlear explants cultures. Media control alone did not induce apparent damage on stereocilia bundles, of which F-actin was intensely labeled with TRITC conjugated phalloidin. Three rows of OHCs and a single row of inner hair cells (IHCs) were clearly observed in the phalloidin staining of a media control group. Treatment with cisplatin resulted in destruction of stereocilia bundles and caused the disarray of three rows of OHCs and a single row of IHCs. However, pretreatment with DSE or RA apparently provided protection against cisplatin-induced stereocilia loss in the primary organ of Corti explants (Fig. 1C). We examined the effect of DSE (1 g/kg) or RA (4 mg/kg) on cisplatin-induced hearing loss in 8-week-old C57BL/6. As shown in Fig. 1D, DSE and RA significantly reduced the cisplatin-induced elevation of auditory brainstem response (ABR) thresholds. DSE or RA alone had no effect on ABR thresholds change (data not shown).

### Molecular docking of the RA and caspase-1 interaction

**Figure 2:**
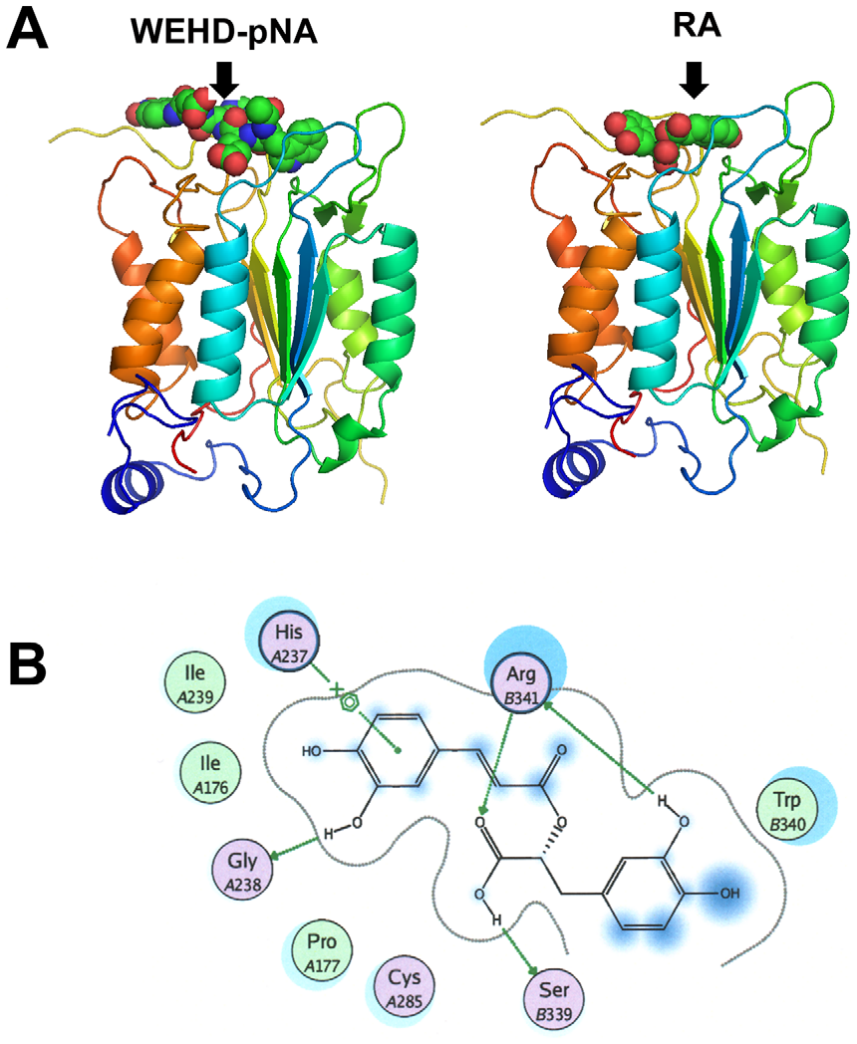
Molecular docking of the RA and caspase-1 interaction. Representative docked poses of the highest binding affinities for the caspase-1 protein with WEHD-pNA and RA (A). Ligand-receptor interaction diagram for the docked pose of RA upon caspase-1 (B). The figure was drawn with MOE modeling package.

To determine whether RA influences to some protein or which protein is a proper receptor for the RA, docking simulations were conducted using candidate proteins. Table S1 summarizes the preliminary docking score of RA with various proteins. RA is known as a specific inhibitor of the Src homology 2 domains of the Src family protein tyrosine kinase. The data for the docking scores indicated that caspase-1 was the best receptor for RA due to its high docking score relative to the other proteins. This computational result was well correlated with experimentally determined Ki values, in which RA ranked the lowest Ki for the caspase-1 protein. The binding mode and affinity of biological substrates and inhibitors for caspase-1 were examined to compare these characteristics with those of RA. The molecular docking results suggested RA is the best ligand for caspase-1. The docking score of caspase-1-RA was 25.01±0.05, which is comparable to the score of 16.77±0.70 for the caspase-1-WEHD-pNA complex (Table 1). The higher docking score of RA indicates stronger binding affinity for caspase-1. The calculated docked structures for the ligands with caspase-1 suggest that WEHD-pNA and RA settle onto an identical binding site of caspase-1 (Fig. 2A). As capase-1 has no deep binding cavity for its ligands on its surface, the ligand-binding process occurs in the shallow crevice on the terminal surface of the receptor protein. The characterized loop structure of caspase-1 wrapping up both WEHD-pNA and RA were observed to form a stable docked complex. In particular, RA showed special inter-atomic interactions, including hydrophobic, electrostatic, and hydrogen bonding (Fig. 2B). The dihydroxyphenyl moiety of RA showed hydrophobic interaction with the His237 residue of caspase-1. The Gly238 and Ser339 residues of caspase-1 were good hydrogen bond acceptors, and Arg341 acted as both a hydrogen bond donor and acceptor for the hydroxyl group of RA. This ligand-bound conformation and the docking score of the RA with caspase-1 provide atomic-level of its inhibitory effect.

**Table 1:** Docking scores of the top-2% ranked poses for complexes between different ligands and the caspase-1 protein.

Ligand	ChemScore	Comment
WEHD-pNA	16.77±0.70	Substrate
YVAD-pNA	22.22±0.37	Substrate
YVAD-CHO	19.05±0.22	Inhibitor
YVAD-CMK	21.36±0.36	Inhibitor
RA	25.01±0.05	

### RA inhibits caspase-1 activation

**Figure 3:**
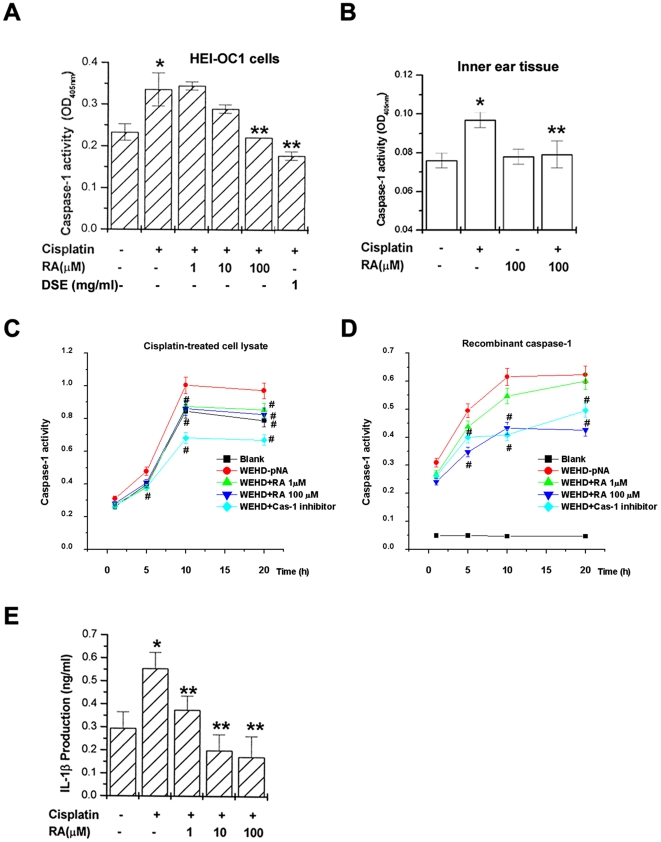
DSE or RA inhibits caspase-1 activation. HEI-OC1 cells (1×10^6^/well) were treated with various concentrations of DSE (1 mg/ml) or RA (1, 10, and 100 µM) for 4 h and then stimulated with cisplatin (20 µM) for 48 h. Caspase-1 activity was determined by a colorimetric kit using substrates in HEI-OC1 cells (A) and inner ear tissue (B). HEI-OC1 cells (1×10^6^/well) were stimulated with cisplatin (20 µM) for 48 h. Catalytic activity of caspase-1 from cisplatin-treated cell lysate (200 µg, C) or recombinant caspase-1 (8 units, D) was measured by WEHD-pNA (caspase-1 substrate), RA (1 and 100 µM), or caspase-1 inhibitor (10 µM) for various times. The color development is monitored at 405 nm (C and D). Each value was calculated from three independent experiments performed in duplicate. IL-1β concentrations were measured in cell supernatants using the ELISA (E). Data are mean ± S.D. of three independent experiments performed in duplicate. *P<0.01, compared with unstimulated cells (determined by independent *t*-test). **P<0.05 compared with cisplatin alone (determined by ANOVA analysis). #*P*<0.05, compared with WEHD-pNA (determined by ANOVA analysis).

Next, we performed the *in vitro* assay based on molecular docking system. Caspase-1 is activated in a variety of cell death paradigms, and it can be activated by treatment with cisplatin [18]. The upstream and downstream mediators of the cell death pathway involving caspase-1 were investigated. To determine if DSE or RA inhibits caspase-1 activation induced by cisplatin, cells were exposed to cisplatin in the presence or absence of various concentrations DSE or RA. Extracts prepared from HEI-OC1 cells exposed to cisplatin contained strong caspase-1 activity compared with unstimulated cells. As shown in Fig. 3A, increased caspase-1 activity was significantly inhibited by treatment with DSE or RA in HEI-OC1 cells. In inner ear tissue, RA also inhibited the caspase-1 activation (Fig. 3B).

A caspase-1 kinetic assay was used to evaluate the binding affinity of RA for the caspase-1 catalytic domain. DSE was not used in the kinetic assay owing to its brown color. Additionally, cisplatin-treated cell lysate and recombinant caspase-1 were also used to confirm the effect of RA in the kinetic assay. As shown in Fig. 3C and 3D, caspase-1 activity was increased after a treatment with WEHD-pNA (caspase-1 substrate), but RA or caspase-1 inhibitor inhibited the binding of WEHD-pNA with the caspase-1 catalytic domain. The inhibition rate by the caspase-1 inhibitor was higher than that of RA in the cisplatin-treated cell lysate (Fig. 3C). Interestingly, RA exhibited a more potent binding affinity than did caspase-1 inhibitor in recombinant caspase-1 (Fig. 3D). This result agreed with molecular docking simulation. Caspase-1 plays a key role in inflammatory responses by cleaving pro-IL-1β and pro-IL-18 into secreted pro-inflammatory cytokines. To investigate the effect of RA on IL-1β production, the downstream mediators of the cell death pathway involving caspase-1, ELISA was performed. As shown in Fig. 3E, RA also significantly inhibited cisplatin-induced IL-1β production.

### RA inhibits expression of apoptotic marker by cisplatin

**Figure 4:**
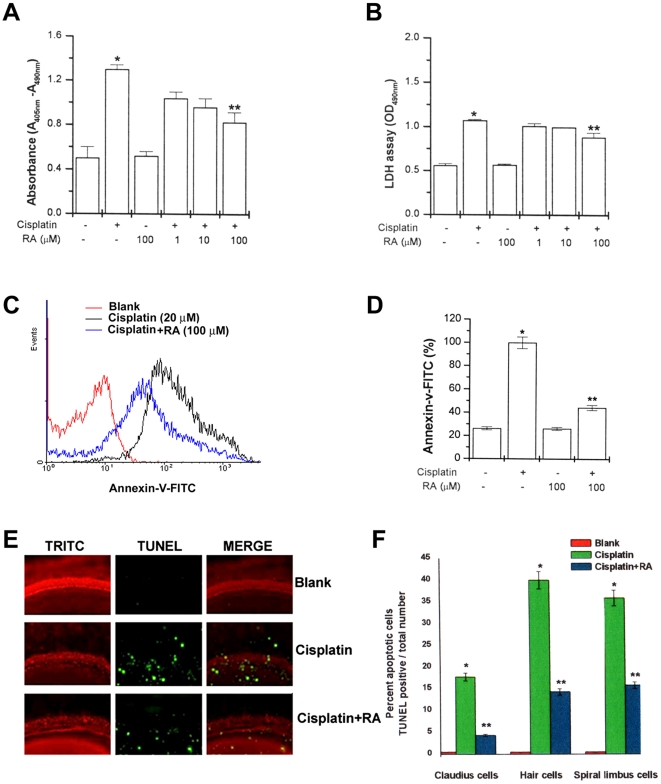
RA inhibits expression of apoptotic marker by cisplatin. HEI-OC1 cells (1×10^6^/well) were treated with various concentrations of RA (1, 10, and 100 µM) for 2 h and then stimulated with cisplatin (20 µM) for 48 h. Internucleosomal DNA fragmentation was quantitatively determined by assaying for cytoplasmic mononucleosome- and oligonucleosome-associated histone accumulated in membrane-intact cells at the indicated time points (A). LDH levels on supernatant were assayed by cytotoxic assay kit (B). Apoptosis was measured by staining with FITC-labeled annexin V, followed by flow cytometric analysis (C), and the percentage of apoptotic cells in the total cell population is shown (D). The basal turns of the cochlea were stained with TRITC-conjugated phalloidin (red), TUNEL (green), and examined under fluorescent microscope. Results are representative of three independent experiments (E). The percentage of apoptotic cells in different type of cells in the explants is shown (F). Data are representative of three independent experiments. Data are mean ± S.D. of three independent experiments performed in duplicate. *P<0.01, compared with unstimulated cells. **P<0.05 compared with cisplatin alone. * and ** represent significance determined by independent *t*-test.

RA revealed a protective effect on cisplatin-induced ototoxicity. Anti-apoptotic cell-death induced by RA was evidenced by morphological changes of the cells (data not shown). RA significantly inhibited nucleosome-sized DNA fragmentation and LDH production increased by cisplatin (Fig. 4A and 4B). To determine whether the inhibition of apoptosis by RA, the early translocation of phosphatidylserine from the internal to external leaflet, a hallmark of early apoptosis, was investigated by incubating HEI-OC1 cells with RA. Cisplatin increased the annexin-V binding. Prior treatment of HEI-OC1 cells with RA blocked annexin-V binding 24 h after the cisplatin treatment (Fig. 4C and 4D).

Subsequently, the effect of RA on the apoptosis of various cells was tested using cisplatin in cochlear explant cultures. The organ of Corti was isolated from rat cochlea on postnatal day 2 and treated with RA and cisplatin for 30 h. TRITC-conjugated phalloidin was used to stain the hair cells, Claudius cells and spiral limbus cells in the cochlear explant cultures. Terminal deoxynucleotidyl transferase dUTP nick end labeling (TUNEL) staining (green) was used to detect apoptosis. The cisplatin treatment destroyed the orderly arrangements of the three rows of OHCs as well as a single row of IHCs and induced apoptosis in the hair cells, Claudius cells, and spiral limbus cells. As shown in Fig. 4E, RA inhibited cisplatin-induced apoptosis. Fig. 4F presents the percentage of apoptotic cells in the different type of cells in the explants.

### RA blocks caspases activity and mitochondrial apoptotic pathways

**Figure 5:**
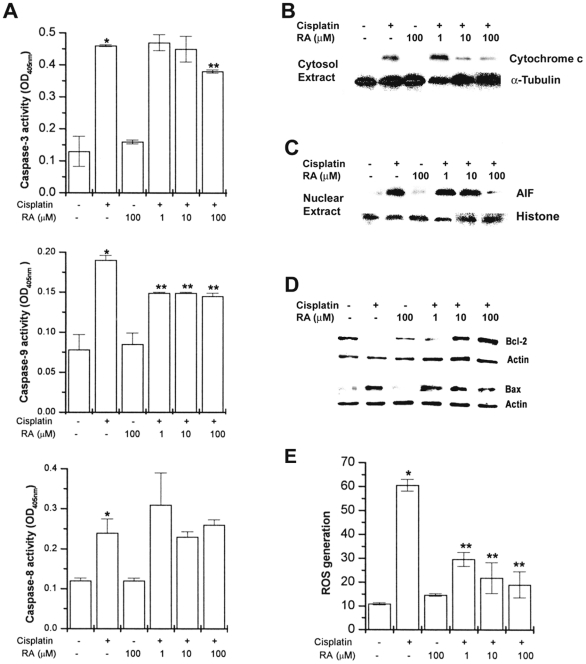
RA blocks cisplatin-induced caspases activation, cytochrome *c* release, AIF translocation, and ROS generation. HEI-OC1 cells (1×10^6^/well) were treated with various concentrations of RA (1, 10, and 100 µM) for 2 h and then stimulated with cisplatin (20 µM) for 48 h. Caspase-3, caspase-8, and caspase-9 activities were determined by a colorimetric kit using substrates (A). Cytochrome *c* release (B) into cytosol, AIF translocation to nucleus (C), and Bax/Bcl-2 (D) were determined by Western blot analysis. HEI-OC1 cells (1×10^4^/well) were treated with various concentrations of RA (1, 10, and 100 µM) for 2 h. ROS generation was increased 24 h after cisplatin treatment (E). Results are representative of three independent experiments. Data are mean ± S.D. of three independent experiments performed in duplicate. *P<0.01, compared with unstimulated cells (determined by independent *t*-test). **P<0.05 compared with cisplatin alone (determined by ANOVA analysis).

Caspase-3, caspase-8, and caspase-9 activity is known to increase in cisplatin-induced apoptosis in HEI-OC1 cells [19]. To determine if RA inhibits caspase activation induced by cisplatin, cells were exposed to cisplatin in the presence or absence of various concentrations RA. Extracts prepared from HEI-OC1 cells exposed to cisplatin contained strong caspase-3, caspase-8, and caspase-9 activity compared to unstimulated cells. As shown in Fig. 5A, increased caspase activities were significantly inhibited by treatment with RA. However, RA did not inhibit cisplatin-induced caspase-8 activation.

The mitochondrial apoptotic cascade requires the release of intermitochondrial membrane cytochrome *c* to the cytosol [20]. Cytochrome *c* released from mitochondria into cytosol was evaluated by a Western blot analysis. The Western blot analysis indicated that the cisplatin treatment induced a cytochrome *c* release and that this was inhibited by treatment with RA (Fig. 5B). As shown in Fig. 5A, RA did not completely inhibit the cisplatin-induced caspase activation. As AIF is known to be involved in apoptosis through a caspase-independent pathway, the possibility that RA inhibits cisplatin-induced AIF translocation into the nucleus was examined. Cisplatin was found to induce mitochondrial AIF release and translocation to the nucleus. RA prevents cisplatin-induced AIF translocation (Fig. 5C). Lee et al. reported that RA effectively suppressed the up-regulation of Bax and down-regulation of Bcl-2 in neuronal cells [21]. As shown in Fig. 5D, RA also regulates the Bax and Bcl-2 expression in cisplatin-induced HEI-OC1 cells.

The effect of cisplatin on intracellular ROS generation was then assessed. Cells were treated with cisplatin for 24 h. The level of intracellular ROS was then monitored using a spectrofluorometer with a peroxide-sensitive fluorescent probe, 2′, 7′-dichlorofluorescein diacetate (DCFH-DA). As shown in Fig. 5E, treatment with cisplatin significantly increased the generation of intracellular ROS. It was found that RA significantly inhibited intracellular ROS generation by cisplatin in a dose-dependent manner.

### RA blocks activation of NF-κB by cisplatin

**Figure 6:**
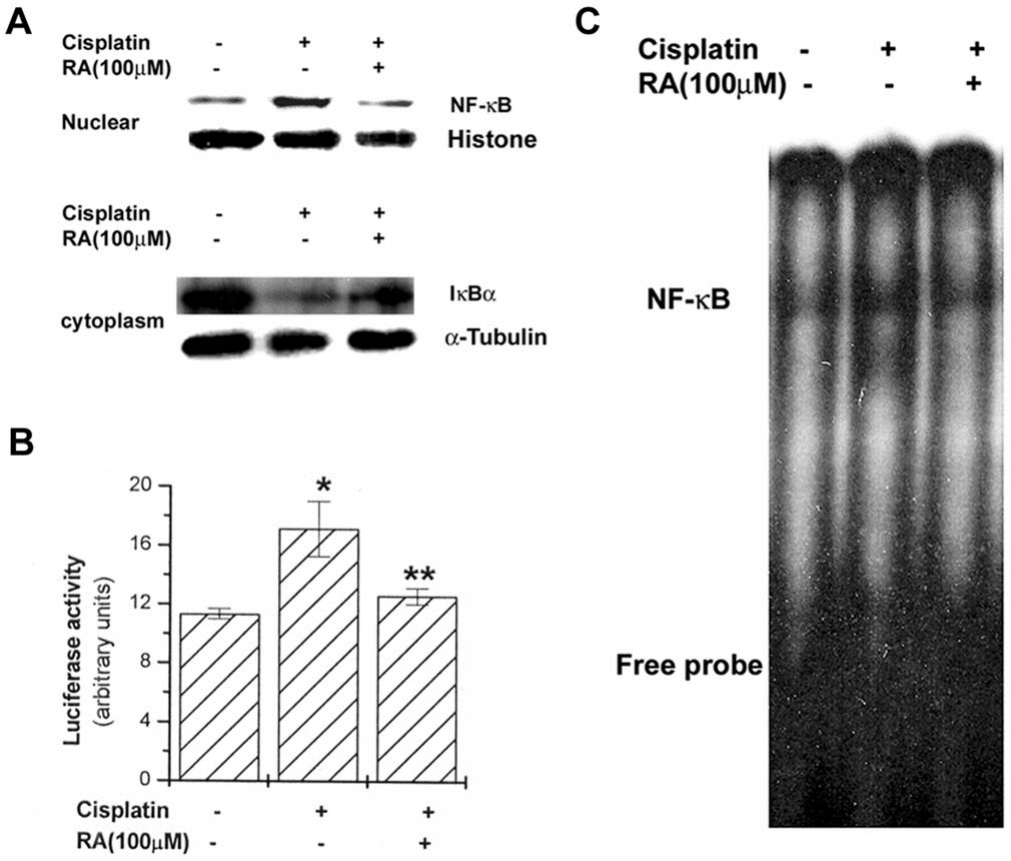
RA inhibits cisplatin-induced NF-κB activation. HEI-OC1 cells (1×10^6^/well) were treated with RA (100 µM) for 2 h and then stimulated with cisplatin (20 µM). Nuclear and cytoplasm protein were prepared and analyzed for NF-κB by Western blotting as described in the experimental procedures (A). The NF-κB activity was assayed by luciferase assay (B). For the electrophoretic mobility shift assay, the nuclear extract incubated with ^32^P-labeled oligonucleotides corresponding to NF-κB was analyzed (C). Data are mean ± S.D. of three independent experiments performed in duplicate. *P<0.01, compared with unstimulated cells. **P<0.05 compared with cisplatin alone. * and ** represent significance determined by independent *t*-test.

As activation of NF-κB is linked to apoptosis, it was assumed that RA mediates its effects at least partly through suppression of NF-κB activation. Treatment by RA (100 µM) inhibited the cisplatin-induced increase of the nuclear NF-κB (*Rel* A/p65) levels in HEI-OC1 cells. As a marker of NF-κB activation, the degradation of IκBα in cell lysates was detected. Activation and nuclear translocation of NF-κB are dependent on the phosphorylation of IκBα, which is then rapidly degraded. It was also shown that RA inhibits cisplatin-induced IκBα degradation (Fig. 6A). To investigate the inhibitory effect of RA on NF-κB activation, we examined the effect of RA in a NF-κB-dependent gene reporter assay. Plasmid NF-κB-luciferase and pSV40-luciferase reporter gene constructs were transiently cotransfected into HEI-OC1 cells, which were stimulated by cisplatin. As shown in Fig. 6B, RA significantly reduced cisplatin-induced luciferase activity. In order to confirm whether NF-κB/DNA binding is inhibited by RA, EMSA were utilized. Treatment of cisplatin increased the DNA binding activity of NF-κB (Fig. 6C), but RA markedly suppressed cisplatin-induced NF-κB/DNA binding activity.

### Effect of RA on cisplatin-treated tumor cells

**Figure 7:**
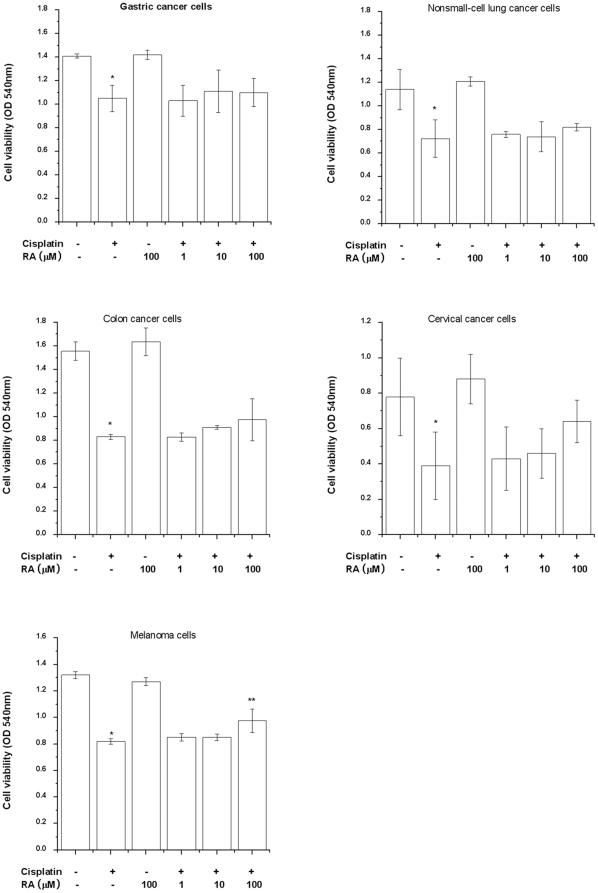
Cancer cells (1×10^5^/well) were treated with various concentrations of RA for 4 h and then stimulated with cisplatin for 96 h. Cell viability was estimated by MTT assay. Data are mean ± S.D. of three independent experiments performed in duplicate. *P<0.01, compared with unstimulated cells (determined by independent *t*-test). **P<0.05 compared with cisplatin alone (determined by ANOVA analysis).

Cisplatin is an anticancer agent. Therefore, the influence of RA on cisplatin-induced anticancer activity was investigated. SNU668 (gastric cancer cell), A549 (non small cell lung cancer cells), HCT116 (colon cancer cell), HeLa (cervical cancer cell), and B16F10 (melanoma cell) cells were pretreated with RA and then stimulated with cisplatin. As a result, cisplatin induced apoptosis of cancer cells. Anticancer activity by cisplatin was not affected by treatment with RA in SNU668, A549, HCT116, and HeLa cells but RA inhibited cisplatin-induced apoptosis in B16F10 cells (Fig. 7).

## Discussion

Cancer chemotherapeutic strategies commonly require multiple agents. However, the use of multiple agents contributes to added toxicity, which often results in poor treatment. Thus, combination chemotherapy must be optimized to increase tumor response and, at the same time, lower the level of toxicity. Natural compounds isolated from plants are used widely in both healthy and diseased humans. Natural compound have many benefits, few (if any) side effects and display low cytotoxicity. This study demonstrated that RA inhibits cisplatin-induced ototoxicity without an effect on the anticancer activity of cisplatin. It also demonstrated that caspase-1 was a target molecule of RA.

Caspase proteases constitute a family of proteases that normally exist as inactive enzymes. These are cystein-dependent, aspartate-specific proteases that function to mediate apoptotic destruction of the cell [22], [23]. Caspases are activated by extrinsic and intrinsic apoptotic pathways [24]. Caspase-1 is an apical caspase in neuronal cell death pathways, mediating Bid cleavage, release of mitochondrial apoptogenic factor (cytochrome c, Smac/Diablo, and AIF), and activation of caspase-3 and caspase-9 [25]. Recent research has shown that ovarian cancer cell death is associated with the activation of caspase-1, caspase-3, and caspase-9 [26]. In inflammatory responses, caspase-1 becomes part of a protein complex, the inflammasome, which has similarities to the apoptosomes in apoptosis [27]. Ghayur et al. reported that caspase-1 was essential for the maturation of the pro-inflammatory cytokines pro-IL-1β and IL-18 in lipopolysaccharide-activated monocytes, as their mature forms are completely absent from monocytes derived from caspase-1-deficient mice [28]. Caspase-1 also plays a role in the activation of NF-κB, as supported by the results of experiments using monocytes derived from caspase-1-deficient mice [23]. The present study showed for the first time that RA inhibited cisplatin-induced caspase-1 activation in HEI-OC1 cells. This suggests that RA inhibits cisplatin-induced apoptosis via a blockade of the caspase-1 pathway.

RA processed anti-oxidant activity as well as anti-inflammatory activities *in vitro* and *in vivo* studies [29], [30]. RA suppressed synivitis in a murine collagen induced arthritis model [31]. Further, RA has anticarcinogenic effects in a murine, two-stage skin carcinogenesis model by inhibiting the inflammatory response and scavenging of reactive oxygen radicals [14]. RA also protected human hepatoma cells (HepG2) from aflatoxin B1- and ochratoxinmediated cell damage [32]. RA was shown to inhibit H_2_O_2_-induced cell injury by its anti-apoptotic and anti-oxidant activity [33]. Recently, RA protects PC12 cells from amyloid-beta peptide-induced neurotoxicity [34]. In our study, RA inhibited cisplatin-induced apoptosis in melanoma cells whereas did not affect cisplatin-induced apoptosis in SNU668, A549, HCT116, and HeLa cells. From this, we presuppose that the protective effects of RA against cisplatin show difference according to cancer cell types. However, further study is necessary to clarify the reaction mechanism between cisplatin and RA in various cancer cells prior to clinical use in patients.

In this study, we showed that RA provided protection against cisplatin-induced stereocilia loss in the primary organ of Corti explants. Thus far, effective research regarding natural compound is attaining much, but research has scarcely been able to demonstrate a feasible effect because compound interacts with certain proteins. In this research, it was found via *in silico* simulations that RA combines with caspase-1. The molecular docking results suggested that RA is the best ligand for the caspase-1. Caspase-1-RA interaction was also confirmed via a kinetic assay. This evidence indicates that the interaction between RA and caspase-1 can explain the diverse pharmacological activities of RA. These findings may also lead to development of more efficacious therapeutics for hearing loss.

In summary, we have demonstrated that treatment of RA inhibited cisplatin-induced apoptosis via inhibition of ROS generation, cytochrome *c* release, caspase activation, AIF translocation, Bax up-regulation, Bcl-2 down-regulation, NF-κB activation, and IL-1β production. In this respect, RA may be attributed to decrease the ototoxic side effect of cisplatin in human being. Further investigation is necessary to determine other possible anti-apoptotic mechanisms of RA and to apply it clinically in ototoxicity environments.

## Materials and Methods

### Ethics statement

All protocols were approved by the institutional animal care and use committee of Kyung Hee University (Protocol Number. KHUASP (SE)-10-036).

### Materials

DMEM and fetal bovine serum (FBS) were purchased from Gibco/BRL (Grand Island, NY, USA). Rosmarinic acid, cisplatin, DMSO, and MTT were purchased from Sigma Chemical (St. Louis, MO, USA). Caspase-1, cytochrome *c*, NF-κB, IκBα, Bax, Bcl-2, and AIF antibodies were obtained from Santa Cruz Biotechnology (Santacruz, CA, USA). Caspase assay kit, recombinant caspase-1, anti-IL-1β antibody, biotinylated IL-1β antibody, and recombinant IL-1β were purchased from R & D system Inc. (Minneapolis, MN, USA). Cytotoxicity detection kit was purchased from Promega (Medison, WI, USA).

### Preparation of DSE

DSE was prepared by decocting the dried prescription of herbs with boiling distilled water (50 g/l). The duration of decoction was about 3 h. The decoction was filtered, lyophilized, and kept at 4°C. The yield of extraction was about 10% (w/w). The DSE water extract powder was dissolved in sterile water (50 g/l). The ingredients of 50 g DSE include 40 g of *Salvia miltiorrhiza* Bunge, 5 g of *Santalum album*, and 5 g of *Amomum villosum* LOUR. The plants materials were obtained from Oriental drug store, NOA (Seoul, Republic of Korea) and identified by H.M. Kim, College of Oriental Medicine, Kyung Hee University. Their voucher specimens (KH090910) have been deposited at the Herbarium at the College of Oriental Medicine, Kyung Hee University.

### Cell viability

HEI-OC1 cells [35] and cancer cells were seeded in 4-well plates (1×10^5^ cells) and exposed to various concentrations with cisplatin. The cell survival fraction was determined with the MTT assay. Absorption was measured by spectrometer at 540 nm.

### Culture of the organ of Corti explants

Spague Dawley rat was sacrificed on postnatal day 2 (P2) and the cochlea was carefully dissected out. The stria vascularis and spiral ligament were dissected away leaving the organ of Corti. The middle turn of the cochlea was used for further analysis. Cochlea explants were treated with high glucose (4.5 g/l) DMEM containing 10% FBS, 20 mM cisplatin and 100 µM RA and further incubated at 37°C in 5% CO_2_ for 30 h. Specimen was fixed for 15 min in 2% paraformaldehyde in 0.1 M phosphate buffer (pH 7.4) at room temperature. Specimen was rinsed in 0.1 M PBS, then incubated in 0.25% Triton X-100 for 2 min and immersed in TRITC-labeled phalloidin in PBS for 20 min. After three washes with PBS, specimen was examined under fluorescence microscope with appropriate filters for TRITC (excitation: 510–550 nm, emission: 590 nm).

### Preparation of inner ear tissue

Mice (n = 5) were given an ip injection of 2 mg/kg of the cisplatin for 8 days. RA was dissolved in water and administered orally 4 h before the injection of cisplatin. Inner ear was isolated at the 9 days after administration of water or RA. Isolated tissue was homogenized for caspase-1 assay.

### Measurement of ABR

ABR was measured before and 24 h after the final treatment with cisplatin. TDT system 3 hardware and software (Tucker-Davis Technologies, Alachua, FL, USA) were used to obtain ABRs, with 1000 stimulus repetitions per record. Mice were anesthetized with a cocktail of ketamine (40 mg/kg) and xylazine (10 mg/kg) and kept warm with a heating pad during ABR recordings. A subdermal (active) needle electrode was inserted at the vertex and ground and reference electrodes were inserted subdermally in the loose skin beneath the pinnae of opposite ears. Auditory stimuli were recorded in response to 100 µs clicks and 10 ms tone burst with a rise/fall time of 1 ms at 4, 8, 16 and 32 kHz. The sound intensity was progressively decreased in 10-dB steps and 5-dB steps (click). The ABR threshold was defined as the lowest stimulus intensity that produced a replicable waveform response. Weight loss was daily monitored during the eight days after all reagent (cisplatin, DSE, and RA) treatments and was supplied 0.6 ml saline solution (intraperitoneally injection, twice/day) for the protection of death with dehydration.

### Assay of IL-1β production

IL-1β production was measured from cells according to the manufacturer's specification using IL-1β assay kit (R & D system Inc, Minneapolis, MN, USA).

### Molecular docking simulation

Molecular docking simulation has been performed using by GOLD 4.0.1 from Cambridge Crystallographic Data Centre. The molecular model for each receptor protein, Src-SH2, caspase-1, NF-κB (p50 homodimer), NF-κB (p65 homodimer), NF-κB (p50p65 heterodimer), and IκBα was obtained from Protein Data Bank (PDB id 1HCT, 1RWK, 1NFK, 1RAM, 1LE5, and 1IKN, respectively) without further modification. Preliminary docking was performed for the system between RA and each receptor protein using the GOLD program with fast searching option. Following docking simulation was performed for different ligand with the caspase-1 protein using by the GOLD program with a ChemScore function. Global searching was performed using a Lamarckian genetic algorithm with a maximum number of 1×10^6^ energy evaluations and 200 individual populations. Other parameters were applied with a mutation frequency of 95, a crossover frequency of 95, and a migration frequency of 10. Independent LGA jobs were carried out for each ligand with 300 parallel runs. Binding affinities were calculated from this 200-pose cluster using a ChemScore Fitness function.

### Cell death assessment by DNA fragmentation assays

DNA fragmentation was measured from cells according to the manufacturer's specification using DNA fragmentation assay kit (Cell Death Detection ELISA plus kit; Roche Molecular Biochemicals, Mannheim, Germany).

### Annexin V-FITC assay

Apoptosis was determined from cells according to the manufacturer's specification using annexin-V-FITC staining kit (invitrogen, Eugene, Oregon, USA). The percentage of cells apoptosis was calculated using the Cellquest software (BD Biosciences, Franklin Lakes, NJ, USA).

### TUNEL staining

Apoptosis was detected by TUNEL technique (In Situ Cell Death Detection Kit, Roche Applied Science, Germany) according to manufacturer's instructions.

### Caspase assay

Caspase activity was measured according to the manufacturer's specification using caspase assay kit (R & D system). A recombinant caspase-1, caspase-3, 8, and 9 enzymes are available for use as a positive control.

### Measurement of intracellular ROS generation

The intracellular ROS level was measured using a fluorescent dye, DCFH-DA (Eastman Kodak, Rochester, NY). In the presence of an oxidant, DCFH is converted into the highly fluorescent DCF. Cells were washed twice with serum-free medium without phenol red and incubated with 5 µM DCFH-DA in serum-free medium without phenol for 10 min. After washing twice with serum-free medium without phenol, the fluorescent intensity was measured at excitation 485 nm and emission 538 nm in a spectrofluorometer.

### Transient transfection and a luciferase assay

For the transfection, we seeded the HEI-OC1 cells (1×10^7^) in a 100 mm culture dish. We then used Lipofectamine™ 2000 (Invitrogen, Carlsbad, CA, USA) to transiently transfect pNF-κB-LUC and pSV40-LUC reporter gene constructs into HEI-OC1 cells. Forty eight hours after the stimulation, we harvested the cells and washed them in cold PBS before lysing them in a 500 µl lysis buffer (Dual-Luciferase® Reporter Assay System; Promega Corporation, Madison, WI, USA). To measure the luciferase activity, we used a luminometer (1420 luminescence counter, Perkin Elmer) in accordance with the manufacturer's protocol. The relative luciferase activity was defined as the ratio of *firefly* luciferase activity to *renilla* luciferase activity.

### Statistical analysis

The experiments shown are a summary of the data from at least-three experiments and are presented as the mean ± S.D. Statistical evaluation of the results was performed by independent *t*-test and ANOVA with Tukey post hoc test. The results were considered significant at a value of *P*<0.05.

## Supporting Information

Table S1 **Docking scores of the RA with the proteins using GOLD program.**
(DOCX)

## References

[1] Trimmer EE, Essigmann JM (1999). Cisplatin.. Essays Biochem.

[2] Humes HD (1999). Insights into ototoxicity. Analogies to nephrotoxicity.. Ann NY Acad Sci.

[3] Liu W, Staecker H, Stupak H, Malgrange B, Lefebvre P (1998). Caspase inhibitors prevent cisplatin-induced apoptosis of auditory sensory cells.. Neuroreport.

[4] Cheng AG, Huang T, Stracher A, Kim A, Liu W (1999). Calpain inhibitors protect auditory sensory cells from hypoxia and neurotrophin-withdrawal induced apoptosis.. Brain Res.

[5] Alam SA, Ikeda K, Oshima T, Suzuki M, Kawase T (2000). Cisplatin-induced apoptotic cell death in Mongolian gerbil cochlea.. Hear Res.

[6] Devarajan P, Savoca M, Castaneda MP, Park MS, Esteban-Cruciani N (2002). Cisplatin-induced apoptosis in auditory cells: role of death receptor and mitochondrial pathways.. Hear Res.

[7] So HS, Park C, Kim HJ, Lee JH, Park SY (2005). Protective effect of T-type calcium channel blocker flunarizine on cisplatin-induced death of auditory cells.. Hear Res.

[8] Lee S, Moon SO, Kim W, Sung MJ, Kim DH (2006). Protective role of L-2-oxothiazolidine-4-carboxylic acid in cisplatin-induced renal injury.. Nephrol Dial Transplant.

[9] Thornberry NA, Bull HG, Calaycay JR, Chapman KT, Howard AD (1999). A novel heterodimeric cysteine protease is required for interleukin-1 beta processing in monocytes.. Nature.

[10] Lamkanfi M, Declercq W, Depuydt B, Caspases Los M, Walczac (2003). Their role in Cell death and cell survival..

[11] Pirvola U, Xing-Qun L, Virkkala J, Saarma M, Murakata C (2000). Rescue of hearing, auditory hair cells, and neurons by CEP-1347/KT7515, an inhibitor of c-Jun N-terminal kinase activation.. J Neurosci.

[12] Cardinaal RM, de Groot JC, Huizing EH, Veldman JE, Smoorenburg GF (2000). Cisplatin-induced ototoxicity: Morphological evidence of spontaneous outer hair cell recovery in albino guinea pigs?. Hear Res.

[13] Kim BC, Lee EJ, Park CS, Park CG (2000). The Experimental Study on the Effects of Dansamyeum on Hyperlipidemia.. J Korean Oriental Med.

[14] Huang YS, Zhang ZT (1992). Antioxidative effect of three water-soluble components isolated from Salviae miltiorrhizae in vitro.. Acta Pharm Sin.

[15] Osakabe N, Yasuda A, Natsume M, Yoshikawa T (2004). Rosmarinic acid inhibits epidermal inflammatory responses: anticarcinogenic effect of Perilla frutescens extract in the murine two-stage skin model.. Carcinogenesis.

[16] Takeda H, Tsuji M, Inazu M, Egashira T, Matsumiya T (2002). Rosmarinic acid and caffeic acid produce antidepressive-like effect in the forced swimming test in mice.. Eur J Pharmacol.

[17] Li X, Yu C, Lu Y, Gu Y, Lu J (2007). Pharmacokinetics, tissue distribution, metabolism, and excretion of depside salts from Salvia miltiorrhiza in rats.. Drug Metab Dispos.

[18] Kondo S, Barna BP, Morimura T, Takeuchi J, Yuan J (1995). Interleukin-1 beta-converting enzyme mediates cisplatin-induced apoptosis in malignant glioma cells.. Cancer Res.

[19] Jeong HJ, Kim SJ, Moon PD, Kim NH, Kim JS (2007). Antiapoptotic mechanism of cannabinoid receptor 2 agonist on cisplatin-induced apoptosis in the HEI-OC1 auditory cell line.. J Neurosci Res.

[20] Kuida K, Lippke JA, Ku G, Harding MW, Livingston DJ (1995). Altered cytokine export and apoptosis in mice deficient in interleukin-1 beta converting enzyme.. Science.

[21] Lee HJ, Cho HS, Park E, Kim S, Lee SY (2008). Rosmarinic acid protects human dopaminergic neuronal cells against hydrogen peroxide-induced apoptosis.. Toxicology.

[22] Boatright KM, Renatus M, Scott FL, Sperandio S, Shin H (2003). A unified model for apical caspase activation.. Mol Cell.

[23] Lamkanfi M, Kalai M, Saelens X, Declercq W, Vandenabeele P (2004). Caspase-1 activates nuclear factor of the kappa-enhancer in B cells independently of its enzymatic activity.. J Biol Chem.

[24] Fernández-Luna JL (2007). Apoptosis regulators as targets for cancer therapy.. Clin Transl Oncol.

[25] Zhang WH, Wang X, Narayanan M, Zhang Y, Huo C (2003). Fundamental role of the Rip2/caspase-1 pathway in hypoxia and ischemia-induced neuronal cell death.. Proc Natl Acad Sci.

[26] Feng Q, Li P, Salamanca C, Huntsman D, Leung PC (2005). Caspase-1alpha is down-regulated in human ovarian cancer cells and the overexpression of caspase-1alpha induces apoptosis.. Cancer Res.

[27] Tschopp J, Martinon F, Burns K (2003). NALPs: a novel protein family involved in inflammation.. Nat Rev Mol Cell Biol.

[28] Ghayur T, Banerjee S, Hugunin M, Butler D, Herzog L (1997). Caspase-1 processes IFN-gamma-inducing factor and regulates LPS-induced IFN-gamma production.. Nature.

[29] Exarchou V, Nenadis N, Tsimidou M, Gerothanassis IP, Troganis A (2002). Antioxidant activities and phenolic composition of extracts from Greek oregano, Greek sage and summer savory.. J Agr Food Chem.

[30] Cao H, Cheng WX, Li C, Pan XL, Xie XG (2005). DFT study on the antioxidant activity of rosmarinic acid.. J Mol Str.

[31] Youn J, Lee KH, Wonm J, Huh SJ, Yun HS (2003). Beneficial effects of rosmarinic acid on suppression of collagen induced arthritis.. J Rheumatol.

[32] Renzulli C, Galvano F, Pierdomenico L, Speroni E, Guerra MC (2004). Effects of rosmarinic acid against aflatoxin B1 and ochratoxin A-induced cell damage in a human hepatoma cell line (Hep G2).. J Appl Toxicol.

[33] Gao LP, Wei HL, Zhao HS, Xiao SY, Zheng RL (2005). Antiapoptotic and antioxidant effects of rosmarinic acid in astrocytes.. Pharmazie.

[34] Iuvone T, De Filippis D, Esposito G, D'Amico A, Izzo AA (2006). The spice sage and its active ingredient rosmarinic acid protect PC12 cells from amyloid-beta peptide-induced neurotoxicity.. J Pharmacol Exp Ther.

[35] Kalinec GM, Webster P, Lim DJ, Kalinec F (2003). A cochlear cell line as an in vitro system for drug ototoxicity screening.. Audiol Neurootol.

