# Supplementary material for: Rosmarinic Acid, Active Component of Dansam-Eum Attenuates Ototoxicity of Cochlear Hair Cells through Blockage of Caspase-1 Activity
Source: PLoS One. 2011 Apr 15;6(4):e18815. doi: 10.1371/journal.pone.0018815 (PMC3078149; doi:10.1371/journal.pone.0018815)
Supplement: Table S1 — Docking scores of the RA with the proteins using GOLD program. (DOCX) [file pone.0018815.s001.docx]

**Table S1** Docking scores of the RA with the proteins using GOLD program

|  | **Src-SH2** | **Caspase-1** | **NF-κB**  **(p50 dimer)** | **NF-κB**  **(p65 dimer)** | **NF-κB**  **(p50p65 dimer)** | **IκBα** |
| --- | --- | --- | --- | --- | --- | --- |
| Dock Score | 32.14 | **36.53** | 27.08 | 32.40 | 30.80 | 27.97 |
| Ki value | 57.95 | **34.38** | 134.00 | 59.70 | 64.60 | 135.50 |
